# Influencing factors and countermeasures on intelligent transformation and upgrading of logistics firms: A case study in China

**DOI:** 10.1371/journal.pone.0297663

**Published:** 2024-04-04

**Authors:** Sixia Fan

**Affiliations:** School of Business, Shanghai Dianji University, Shanghai, China; King Khalid University, SAUDI ARABIA

## Abstract

This study explores the influencing factors on intelligent transformation and upgrading of China’s logistics firms under smart logistics, and designs the corresponding framework to guide the practice of firms. By analyzing the characteristics of smart logistics and the transformation and upgrading needs of traditional logistics, from the micro perspective of logistics firms, this paper constructs influencing factor index system of smart transformation and development from four dimensions: logistics technology innovation, logistics big data sharing, logistics management upgrading and logistics decision-making transformation. Logistics firms are divided into firms with medium scale and above and small and medium-sized firms according to their scale. Then EWIF-AHP model is proposed to measure the weight of index system and score the decision-making, so as to evaluate the impact of various influencing factors on transformation and development of logistics firms. The results show that, for logistics firms above medium scale, logistics technology innovation and logistics big data sharing have the most significant impact on transformation and development, followed by logistics management upgrading and logistics decision-making transformation. For small and medium-sized logistics firms, the biggest factor is the upgrading of logistics management, followed by the upgrading of logistics technology, which is almost as important as the influencing factors of the upgrading of logistics management, and followed by the sharing of logistics big data and the transformation of logistics decision-making. Therefore, corresponding countermeasures and suggestions for intelligent transformation of logistics firms have been put forward.

## 1 Introduction

Logistics industry is the basic, strategic and forward-looking industry to support development of national economy, and it is also the pillar industry to carry development of manufacturing, services, industry and agriculture. With blowout development of technological innovation and in-depth refinement of global supply chain, all walks of life are driven by innovation and moving towards the development path of modernization and intelligence [1]. As an accommodation and leading industry, logistics has become the focus of social attention.

The concept of smart logistics was first introduced by the concept of "smart earth" proposed by IBM in 2008. Smart logistics is valued by many countries and governments [2, 3]. Moreover, the realization of smart logistics should rely on the transformation and upgrading of logistics intelligence [4, 5] Intelligent transformation and development of China’s logistics industry is affected by many factors, such as technological innovation, independent core components, management means and other internal factors, as well as industrial structures, government regulations, environmental resources and other external factors [6–8]. The different combinations of these factors will determine the development direction of logistics enterprises. For instance, these could make enterprises having higher operation efficiency and undertaking greater logistics intensity. However, it also faces high facility and equipment costs and the renewal and elimination of existing employees. Therefore, selecting which factors and aspects to upgrade and transform logistics intelligently, in order to improve the enterprises’ market competitiveness could be an urgent problem that enterprises need to solve. Researches on the influencing factors of logistics intelligent transformation mostly focused on the macro level, while researches on the influencing factors with logistics firms as the micro level were less [9, 10]. Meanwhile, the research scope is relatively scattered and mostly concentrating on single point technology in logistics links. However, the logistics industry is composed of many logistics firms. In addition to information infrastructure construction, policy support, market regulation and other factors which led by governments, the intelligent development degree of logistics firms is the core driving force to promote the overall leap forward in logistics industry [11, 12]. Therefore, from the micro perspective of logistics firms, this paper extracts the comprehensive factors affecting logistics intelligent transformation of firms, and evaluates and analyzes the influence ability and action path, so as to guide firm intelligent innovation and intelligent transformation. Additionally, it could boost the overall transformation and upgrading of China ’ s logistics industry and accelerate logistics development.

The informatization of logistics industry has gone through four stages: single point information age, network information age, supply chain interconnection age and intelligent logistics age. Many scholars have studied influencing factors of smart logistics development from macro level and social dimension, and conducted theoretical researches from the definition of smart logistics concept, smart logistics information platform, smart logistics information system application, policy and regional regulation [13–15]. The influencing factors of smart logistics development are the core elements of the emerging trend of industry information development. In terms of smart logistics definition, Nowicka (2014) defined the sustainability of smart logistics from social, economic and environmental dimensions [16]; Bao and Zhang (2014) driven by Internet +, build logistics service system and emphasized the application of artificial intelligence technology and information technology in the field of logistics [17]. In the construction of smart logistics information platform, Qin (2018) put forward the concept of business ecosystem logistics information platform, studied the connotation and development objectives of ecological logistics information platform [18], and also clarified the operation subject, operation mode; Yang et al. (2017) and Sun (2015) proposed a smart logistics platform based on cloud computing, and designed the hierarchical structure of the platform from users group [19, 20], support environment and other modules. In the application of smart logistics information system, Cai (2020) selected evaluation indexes from aspects of system development, construction [21], operation and performance to explore the economy of smart logistics information system and put forward regulation schemes from policies and regions. Wang (2014) analyzed the supporting role of modern and high-level intelligent logistics on urbanization construction [22], from the perspective of the relationship between social logistics and urbanization. Based on the macro perspective of regional smart industry development, Chen and Zhang (2017) proposed to build a smart logistics model with location characteristics, industrial advantages and regional development needs [23].

At present, there are few studies on the influencing factors of smart logistics transformation from the micro perspective of logistics firms in academia. Therefore, this paper summarizes the influencing factors of logistics firms’ intelligent transformation, extracts its core indicators, divides logistics enterprises into two parts according to their scales and evaluates and analyzes its influencing ability and action path by using EWIF-AHP model. Then, build factors system architecture of logistics firms’ smart development. It could provide an evaluable theoretical framework for intelligent transformation of firms, and promote the overall smart transformation and development of the logistics industry.

## 2 Factors analysis and index system of logistics firms’ intelligent development

Smart logistics is a high-level stage of information construction, which focuses on the development of logistics data perception automation, diversified calculation and analysis, modular information processing, intelligent manipulation and control, and resource sharing and interworking. Smart logistics has the characteristics of science and technology intensive, and its development level depends on logistics firms informatization levels and the application and popularization of logistics technologies and equipment. In the process of intelligent transformation, logistics firms will mainly focus on the innovation and development of logistics technology, the interconnection and sharing of logistics big data, the upgrading and transformation of logistics management and the intelligent adjustment of logistics decision-making, which are the core areas of the whole logistics industry intelligent development. Therefore, this paper extracts and analyzes the main factors affecting logistics firms’ intelligent transformation, based on the characteristics of logistics intelligence, the current situation of China’s logistics firms, the characteristics of modern logistics operation environment and other factors.

### 2.1 Influencing factors analysis

#### 2.1.1 Logistics technology innovation ability analysis

Traditional logistics firms are small in scale and numerous in quantity, with labor-intensive and manual operation as the main operation mode. However, in the face of the rapid development of national economy and the continuous upgrading of consumption quality and quantity, traditional logistics technology, equipment and mode are unsustainable. Logistics technology and equipment are the direct carrying poles that contacting goods and personnel, and are the evaluation indicators that determining the logistics firm’s throughput capacity. At the same time, the innovation and upgrading of logistics technology and equipment directly affect firms’ development scale and business scope. Therefore, firms should subdivide logistics technology and adjust the innovation investment and transformation at the levels one by one.

*a*. *Perception technology equipment investment degree*. Logistics perception technology is the basic technology of logistics operation framework. It mainly focuses on RFID, QR code, sensor, reader, NFC, GPS, GIS and other technologies, and completes the goods information collection and transmission through multiple sensing devices [24–27]. Traditional logistics pays more attention to the delivery of goods and less attention to the information which collected in the process of transportation. While, smart logistics extends the goods delivery from a point on the two-dimensional plane to the tracking, monitoring and feedback of the good’s whole life cycle. Thus, if firm wants to master the real-time information of the whole process, cycle and life of goods, it needs to improve the investment and transformation of perception technology and equipment.
*b*. *Connecting technical equipment investment degree*. Logistics connection mainly focuses on handling, loading and unloading and sorting, which is the connecting step of various logistics operation links. Traditional logistics firms mostly take eye recognition, manual handling and manual sorting as the operation mode. However, the quantity of modern logistics freight goods is huge, the types are complex and the batch is dense. Therefore, in order to shorten the freight turnover period and reduce the logistics cost, the traditional logistics firms are unable to cope with this kind of logistics reform. Smart logistics advocates logistics digitization, automation and high efficiency, mainly with intelligent equipment such as stacker, ASRS, shuttle, AGV, automatic sorting machine [28, 29]. Logistics firms need to increase the investment in automation and intensification of logistics connection equipment, improve the automation level of connection equipment, and adjust it to firm operation mode of replacing people with machines and human-machine symbiosis.
*c*. *Mobile technology equipment investment degree*. Mobile technology equipment is the core component of logistics transportation and delivery, which can be divided into mobile technology and intelligent transportation equipment from the technical level. With the popularization of mobile terminals and the nested application of technologies such as CNF, GPS and mobile payment, the new logistics mobile technology can realize element interconnection and information intercommunication [30]. POS machine, scanner, smart phone, PAD and other machine become the smart logistics mobile carriers. On the other hand, mobile transportation equipment is also developing towards more automatic, intelligent and unmanned control, with the globalization of commodity transportation area, the complexity of transportation operation scope and the intensification of transportation batches. More and more unmanned vehicles, unmanned drones, unmanned underwater vehicles are used in the logistics industry, so that employees are far away from complex terrain and dangerous working environment. Therefore, the degree of investment in mobile technology and equipment can affect transportation limits and efficiency of logistics operations, and also can determine the number and distribution of logistics participants.
*d*. *Infrastructure upgrades level*. Logistics infrastructure is the core factor to determine the logistics sustainable development. In addition to macro-government construction such as transportation, shipping, road network and underground engineering, the micro-space area which connecting intelligent technology and equipment is another key issue that firms need to focus on. The traditional logistics firm infrastructure is mostly self-built or leased simple factory buildings. Additionally, the structure is simple, and easy to build artificial shelves. However, the modern logistics operation requires the rapid warehousing, outbound, sorting, inspection and other operations of goods. It is difficult to meet the rapid circulation speed only by manual entry operation. The application of artificial intelligence needs the cooperation of auxiliary facilities, such as the construction of Zigbee, 6G/5G/4G and other multi-network, the establishment of a variety of wireless charging methods such as inductance and magnetic coupling, and the construction of flexible space blocks to adapt to ASRS and multi-terminal storage mode. By improving the advanced and intelligent logistics infrastructure, artificial intelligence can promote the efficient operation of intelligent equipment in logistics firms.

In view of this, this paper selects perception technology equipment investment degree, connecting technical equipment investment degree, mobile technology equipment investment degree and infrastructure upgrades level as the evaluation indexes of logistics technology innovation ability to measure the hardware levels in intelligent transformation of logistics firms.

#### 2.1.2 Logistics big data sharing ability analysis

Logistics is a social and economic activity that integrates multiple functions, modules, operation modes and multiple participants. The industry itself has a huge scale of data. Some of these data come from corporate customers and business processes, and the other part comes from the circulation information of goods in supply chain. Data are gathered in the dimensions of link, time and space, so the magnitude of data will make a qualitative leap. Compared with traditional logistics, smart logistics pays more attention to the high-speed acquisition, processing, analysis, transmission and sharing of data. Except that advanced logistics technology and equipment can achieve rapid data collection, the transmission and application of logistics big data have become the key factors affecting the development scale and operation efficiency of firms [31].

*a*. *Application level of internal information platform*. Since the 1980s, with the development of logistics information, traditional logistics firms have gradually changed from manual accounting to Excel form using, to the application of a single logistics module small program, and then to the access of ERP, MRP and other systems [32, 33]. The ability of data reading and information transmission analysis of the whole process has become one of the core evaluation indexes of firm’s logistics operation rate. The application level of internal information platform can visually describe the proportion of information records in logistics links, subjects, goods and other aspects of enterprise operation. Additionally, the higher the proportion is, the higher the level of information platform application is, and the more the share of big data is. Therefore, firms should refine their internal operations, build information modules one by one, and jointly build information platforms.
*b*. *Compatibility level of external system*. Logistics is used to realize the movement of goods within the scope of space. There are many operating technologies, equipment and facilities involved in the process of movement. Information and data need to be read and transferred between multiple devices and platforms. The compatibility of external systems directly affects the normal acquisition of data and information. Therefore, logistics firms should pay attention to the smoothness of connection and information communication between platforms when designing, developing and applying logistics information technology and platforms. On the other hand, with the popularity of smart phone terminals, information is transferred from a fixed client to a mobile client without space and time constraints, and information acquisition is transformed from wired to infrared, and then to the current wireless, 5G and other multi-network communication systems. Firms’ logistics data storage and transmission need to cross the field equipment, remote equipment, mobile equipment and other multi-regional, multi-level, multi-agent system platform. Therefore, the compatibility level between external systems directly affects the accuracy of information transmission, reflecting whether firms have international versatility in the selection of information system architecture.
*c*. *Data standardization degree*. Smart logistics puts forward the goal of “accelerating intensive integration and promoting the co-construction and sharing of logistics big data resources”. Logistics data come from multiple links and modules. The acquisition subjects such as acquisition components, facilities and equipment, and information platforms are various, and the information processing and storage rules are also different. However, the premise of data sharing is based on the connectivity and interpretability of data. Differences in data elements and their format types can lead to redundant data storage, data interpretation deviation or failure, and poor information transmission and distortion. At the same time, the global supply chain requires the joint participation of multiple firms and multiple subjects. The error of data in sharing and transmission will lead to the superposition of information error rate and the bullwhip effect, which is not conducive to the normal operation of the supply chain. Thus, the degree of data standardization reflects the storage and sharing of data between firms and firms, firms and industries, industries and industries, industries and regions, countries, and even international.
*d*. *Information interconnection investment*. Smart logistics emphasizes data sharing. The prerequisite of data sharing is the configuration of data storage container and sharing channel. The storage container is the representation of resource storage space. The larger the space is, the larger the storage capacity is, and the more resources that firms can call. The introduction of cloud and blockchain has gradually transformed logistics into the integration mode of the Internet of Things, cloud and blockchain [34–36]. This mode is not only distributed storage space, decentralized, but also is the loose storage architecture, the flexibility of information connection calls. So, traditional logistics firms from C/S mode, into a more efficient information processing, resource-free temporary storage B/S mode. The configuration of shared channels depends more on the network environment. The advancement of network infrastructure and the versatility of network protocol architecture directly affect the accessibility of information transmission. Information interconnection investment is used to measure the basic condition level of firm logistics information sharing and evaluate its collaborative information ability. Considering this, application level of internal information platform, compatibility level of external system, data standardization degree and information interconnection investment are selected as the evaluation indexes of logistics big data sharing ability, which can be used to measure the level of information development in intelligent transformation of logistics firms.

#### 2.1.3 Logistics management upgrade ability analysis

Logistics management can effectively organize, plan, operates, command and control enterprise logistics activities, and it can be conducted in-depth regulation from aspects of technology, capital and human resources. Smart logistics introduces artificial intelligence into traditional logistics, and puts forward higher requirements from personnel allocation, management mode, participation subject and other factors.

*a*. *High-skilled personnel input*. Traditional logistics is characterized by labor-intensive industrial structure, and employees are mainly middle- skilled or low- skilled labor, and engaged in regular repetitive physical labor. According to Rolandberg survey report, the rapid development of robotics in the field of logistics, in the next 20 years, Europe will face the loss of nearly 10,000 low-skilled logistics employees, and the number of lost jobs will reach 1.5 million. The introduction of new technologies will lead to redefinition of nearly 3.6 million logistics jobs, and a large number of new skilled employees will flow into logistics market. Logistics firms will also face this kind of change in China. The input of high-skilled personnel is one of the flexible indicators to measure the synergy between enterprise employees and new technology development, whether it has man-machine symbiosis and adapts to industry development. On the one hand, it can evaluate the level of enterprise automation; on the other hand, it can evaluate the efficiency of enterprise logistics operation.
*b*. *Shared resource balancing level*. In traditional logistics, shared resources are mainly based on the resources of third-party logistics firms. Through simple logistics activities such as distribution and warehousing, the demand balance between supply and demand is stimulated. Under smart logistics, resource sharing is not only material resource sharing, but also human resource sharing, technical information sharing and data platform sharing. Under COVID-19, many logistics firms start employee sharing mechanism, taking the region as the unit, and logistics employees mainly participate in logistics work by random secondment. In response to this new logistics management mode, firms not only need to re-plan the type, quantity and skill level of employees, but also need to dynamically adjust the logistics resources and basic environment so as to adapt to the self-management redundancy or insufficient supply after resource sharing. The level of shared resource balance can evaluate the balance state of firms in planning and adjustment, so as to evaluate the flexibility and sustainability of firms.
*c*. *The combination degree of industry*, *politics*, *education and research*. Intelligent logistics leads firms to realize intelligence step by step. To make best use of artificial intelligence facilities and equipment in firms. More flexible and efficient logistics operations, advanced management tools, intelligent machine learning algorithms, reasonable outdoor operating environment and other factors need to be integrated into firm to improve the performance of firm’s sustainable development. On the one hand, these factors come from firms’ own experience accumulation and peer learning; on the other hand, it comes from knowledge sharing among government, universities and research institutes. Therefore, high-skilled personnel input, shared resource balancing level, and the combination degree of industry, politics, education and research are selected as the evaluation indexes of logistics management upgrading ability to measure soft power development level of logistics firms’ intelligent transformation.

#### 2.1.4 Logistics decision-making transformation ability analysis

Logistics decision-making is the top-level module of logistics operation framework. Intelligence of its decision-making ability affects overall intelligent development level of logistics operation.

*a*. *Logistics demand level*. Logistics decision-making is divided into business layer, tactical layer and strategic layer according to vertical extension of its decision-making level. Degree of intelligent development of each layer depends on the level of logistics demand. Business layer is at the basic level of logistics decision-making, and the increase of logistics demand leads to the increase of the input of artificial intelligence facilities and equipment. Intelligent equipment modules can gradually replace human decision-making to realize automation and intelligence of business process decision-making. With popularization of Internet technology, the rapid development of e-commerce mode and the rapid growth of logistics demand, the change of demand is not only the change of online and offline mode, but also the transformation and transformation of regional and national geographical space. In response to this kind of market change, logistics strategy and tactical decision-making need to quickly improve its intelligent decision-making level. Using logistics big data platform and advanced learning evolution algorithm, accurately and quickly predict market changes and industry needs, deploy and plan its development model, hardware holdings and supporting human capital as soon as possible, and form intelligent and intelligent logistics decision-making from top to bottom according to the level of logistics demand.
*b*. *Policy guidance promotion*. Intelligent transformation of firm’s logistics cannot be separated from the governments and regions promotion and support. Intelligence of infrastructure and digitization of information platform are the primary conditions for firms to face under the external living environment. Preferential policies and subsidies of governments can attract logistics firms to settle down. However, in order to develop rapidly and adapt to the new changes of intelligent logistics, government needs to make a long-term plan, with new regional market mechanisms, market development strategies, industrial supporting patterns, human supply structures, ecological resources and other policies. Based on this, firms dynamically adjust their decision-making and development, which integrated with governments and regions, and realize a two-way win-win model.
*c*. *Innovative driving degree of new logistics mode*. Cloud technology, Internet +, blockchain, crowdsourcing, cross-border and other new technologies are integrated into logistics industry, which is a new model for logistics intelligent development. Logistics is not only applied to service industry, but also to agriculture and industry. With the development of industry 4.0, the deepening of Made in China 2025 and the implementation of agricultural supply-side reform, logistics industry, as one of the core industries of the tertiary industry, should be coordinated with industry and agriculture [37, 38]. Consequently, logistics demand level, policy guidance promotion and innovation driven degree of new logistics mode are selected as the evaluation indexes of logistics decision-making ability to evaluate the generalization performance and extension performance of logistics enterprise intelligent transformation.

### 2.2 Index system

By analyzing characteristics of logistics intelligence, current situation of logistics firms in China, modern logistics operation environment and other elements, the influencing factors of transformation and development of logistics firms in China have been summarized. The primary indicators of influencing factors are defined as four indicators: logistics technology innovation, logistics big data sharing, logistics management upgrading and logistics decision-making transformation.

**Fig 1:**
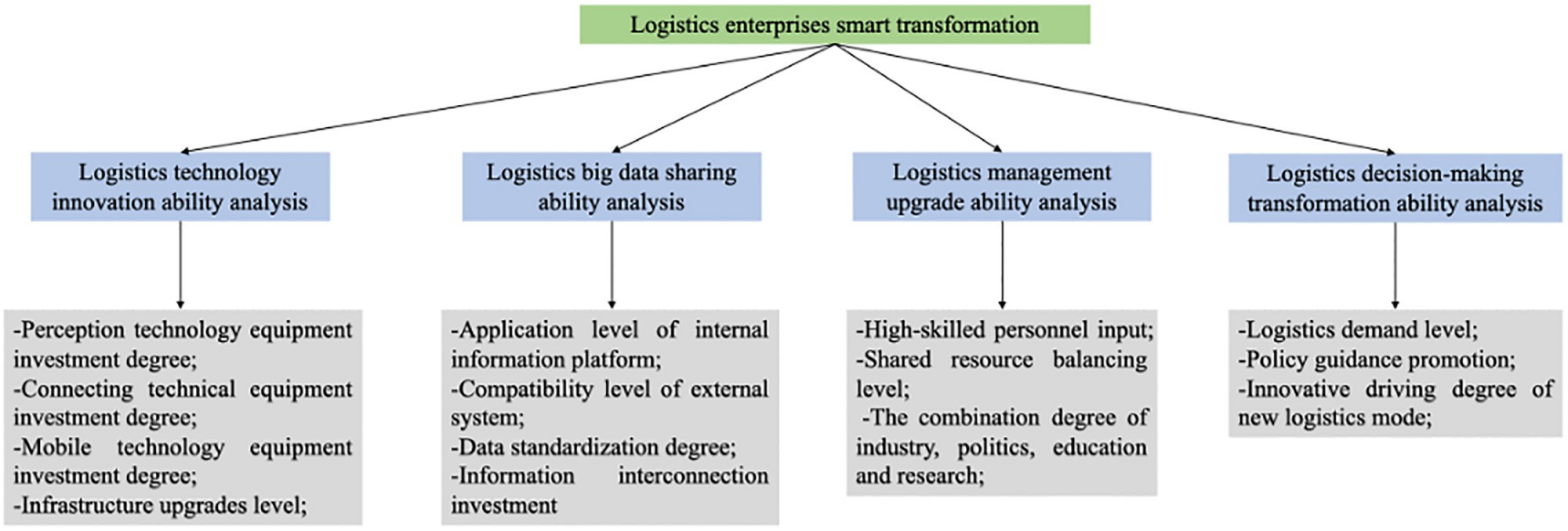
Index system framework of influencing factors on logistics firms’ intelligent transformation.

As shown in Fig 1, the secondary indicators of logistics technology innovation are perception technology equipment investment degree, connecting technical equipment investment degree, mobile technology equipment investment degree and infrastructure upgrades level; the secondary indicators of logistics big data sharing are application level of internal information platform, compatibility level of external system, data standardization degree and information interconnection investment; the secondary indicators of logistics management upgrading are high-skilled personnel input, shared resource balancing level and the combination degree of industry, politics, education and research; the secondary indicators of logistics decision-making transformation are logistics demand level, policy guidance promotion and innovative driving degree of new logistics mode.

## 3 Influencing factors evaluation model of logistics firms’ intelligent transformation and development

Based on the above index system architecture, Entropy Weight Intuitionistic Fuzzy (EWIF) evaluation method has been used to construct the influencing factors evaluation model of logistics firms’ intelligent transformation and development.

### 3.1 EWIF-AHP evaluation model

Analytic Hierarchy Process (AHP) is a quantitative and qualitative comprehensive evaluation method proposed by Saaty (2000) [39]. According to understanding of program objectives, decision makers or decision experts make corresponding decisions by using preference order, utility value or preference relationship to evaluate. Due to the prior limitation of expert evaluation, AHP can only judge two extreme cases, while fuzzy dynamic judgment of environment is lacking. Van Laarhoven and Pedrycz (1983) integrated fuzzy mathematics into AHP, and Buckley established Fuzzy AHP (FAHP) evaluation method on this basis [40]. On the basis of fuzzy set theory, Atanssov (1986) theoretically extended it and derived intuitionistic fuzzy sets. Intuitionistic fuzzy sets make up fuzzy sets which can only divide expert decision-making state into membership and non-membership state, but establish membership, non-membership and hesitation three modes. Entropy originates from information theory [41], and objectively evaluates its value according to the order of magnitude of each information transmitted to decision-making experts, so as to obtain the weight. That is the assignment concept of entropy weight. Then, entropy weight assignment is introduced into the intuitionistic fuzzy theory, and the expert evaluation fuzzy set is transformed by entropy weight to make its evaluation judgment more realistic and flexible. Therefore, this paper combines Entropy Weight Intuitionistic Fuzzy with AHP to structure EWIF-AHP model. Then, use EWIF-AHP to analyze the influencing factors of logistics firms’ intelligent transformation in China.

Definition 1: Set the intuitionistic fuzzy set (IFS), assuming that the non-empty set *X*, and then

$$A=\{<x,\mu_{A}\left(x\right),\nu_{A}\left(x\right)>|x\in X\},X=\left\{x_{1},x_{2},\ldots ,x_{n}\right\}.$$

*A* is the intuitionistic fuzzy set composed of three elements, *μ*_*A*_(*x*) is the membership degree of *x* (internal factor of *X*) belonging to *X*; *ν*_*A*_(*x*) is the non-membership degree of *x* (internal factor of *X*) in the field of *X*.

$$\mu_{A}:\;X\to \left[0,1\right],\;x\in X\to \mu_{A}\left(x\right)\in \left[0,1\right],$$

$$\nu_{A}:X\to \left[0,1\right],x\in X\to \nu_{A}\left(x\right)\in \left[0,1\right]$$

and the conditions of 0 ≤ *μ*_*A*_ + *ν*_*A*_ ≤ 1 and *x* ∈ *X* are met.

Suppose *π*_*A*_(*x*) = 1 − *μ*_*A*_(*x*) − *ν*_*A*_(*x*), 0 ≤ *π*_*A*_(*x*) ≤ 1, and *π*_*A*_(*x*) is the hesitation degree of *x* (internal factor of *X*) attached to *X*, indicating its undefined level. Intuitionistic fuzzy is a special state when *π*_*A*_(*x*) ≥ 0, and the fuzzy set defined by Zedeh is derived.

Definition 2: Set intuitionistic fuzzy numbers *a* = (*μ*_*a*_, *ν*_*a*_, *π*_*a*_), where *μ*_*a*_ ∈ [0,1], *ν*_*a*_ ∈ [0,1], and then

$$\mu_{a}+\nu_{a}\leq 1,\pi_{a}=1-\mu_{a}-\nu_{a}$$

Definition 3:Set ${\tilde{a}}_{1},{\tilde{a}}_{2}$ be two intuitionistic fuzzy numbers on the domain *X*, where ${\tilde{a}}_{1}={(\mu }_{1},\nu_{1},\pi_{1})$, *μ*_1_ ∈ [0,1], *ν*_1_ ∈ [0,1], and *μ*_1_ + *ν*_1_ ∈ [0,1]; ${\tilde{a}}_{2}={(\mu }_{2},\nu_{2},\pi_{2})$, *μ*_2_ ∈ [0,1], *ν*_2_ ∈ [0,1], and *μ*_2_ + *ν*_2_ ∈ [0,1].

Set *λ* be a real number and *λ* ≥ 0, then the operation rules for defining intuitionistic fuzzy numbers are as follows:

$${\tilde{a}}_{1}⊕{\tilde{\;a}}_{2}=(\mu_{1}+\mu_{2}-\mu_{1}\mu_{2},\nu_{1}\nu_{2})$$ (1)

$${\tilde{a}}_{1}⊗{\tilde{\;a}}_{2}=(\mu_{1}\mu_{2},\nu_{1}+\nu_{2}-\nu_{1}\nu_{2})$$ (2)

$$\lambda {\tilde{\;a}}_{1}=(1-{\left(1-\mu_{1}\right)}^{\lambda },\nu_{1}^{\lambda })$$ (3)

$${\tilde{\;a}}_{1}^{\lambda }=(\mu_{1}^{\lambda },1-{\left(1-\nu_{1}\right)}^{\lambda })$$ (4)

then the average fuzzy numbers of ${\tilde{a}}_{1}\;and\;{\tilde{a}}_{2}$ are $\bar{x}=\frac{1}{2}\left({\tilde{a}}_{1}+{\tilde{a}}_{2}\right)=(1-\mu_{2}{\left(1-\;\mu_{1}-\mu_{2}+\mu_{1}\mu_{2}\right)}^{\frac{1}{2}}),\;{\left(\nu_{1}\nu_{2}\right)}^{\frac{1}{2}})$.

Definition 4: Let $\tilde{A}$ and $\tilde{B}$ to be two intuitionistic fuzzy sets. $\tilde{A}=\{<\mu_{\tilde{A}}(x_{i}),\nu_{\tilde{A}}(x_{i})>|x_{i}\in X\}$ and $\tilde{B}=\{<\mu_{\tilde{B}}(x_{i}),\nu_{\tilde{B}}(x_{i})>|x_{i}\in X\}$. If *IFS*(*X*) → [0,1], then let function E be an intuitionistic fuzzy entropy. And E meets the following criteria.

Criteria 1: If and only if $\tilde{A}$ is a clear set, $E\left(\tilde{A}\right)=0$, and $\mu_{\tilde{A}}\left(x_{i}\right)=1,\nu_{\tilde{A}}\left(x_{i}\right)=0$, or $\mu_{\tilde{A}}\left(x_{i}\right)=0,\nu_{\tilde{A}}\left(x_{i}\right)=1$, when ∀*x*_*i*_ ∈ *X*.

Criteria 2: $E\left(\tilde{A}\right)=1$, if and only if ∀*x*_*i*_ ∈ *X*, then $\mu_{\tilde{A}}\left(x_{i}\right)=\nu_{\tilde{A}}\left(x_{i}\right)$

Criteria 3: $E\left(\tilde{A}\right)=E\left({\tilde{A}}^{c}\right)$, when $\pi_{\tilde{A}}=\pi_{\tilde{B}}=C$.

Criteria 4: *$E\left(\tilde{A}\right)\leq E\left(\tilde{B}\right)$*, if ∀*x*_*i*_ ∈ *X*, then

1. when $\mu_{\tilde{B}}(x_{i})\geq \nu_{\tilde{B}}(x_{i})$, $\mu_{\tilde{A}}\left(x_{i}\right)\geq \mu_{\tilde{B}}(x_{i})$ and $\nu_{\tilde{A}}(x_{i})\leq \nu_{\tilde{B}}(x_{i})$.
2. When $\mu_{\tilde{B}}(x_{i})\leq \nu_{\tilde{B}}(x_{i})$, $\mu_{\tilde{A}}\left(x_{i}\right)\leq \mu_{\tilde{B}}(x_{i})$ and $\nu_{\tilde{A}}(x_{i})\geq \nu_{\tilde{B}}(x_{i})$.

When $\tilde{A}$ and $\tilde{B}$ are two intuitionistic fuzzy sets, then the operation rules for defining intuitionistic fuzzy sets are as follows:

$$\tilde{A}+\tilde{B}=\left(\mu_{\tilde{A}}\left(x_{i}\right)+\mu_{\tilde{B}}\left(x_{i}\right)-\mu_{\tilde{A}}\left(x_{i}\right)\cdot \mu_{\tilde{B}}\left(x_{i}\right),\nu_{\tilde{A}}\left(x_{i}\right)\cdot \nu_{\tilde{B}}\left(x_{i}\right)\right)$$ (5)

$$\lambda \tilde{A}=(1-{\left(1-\mu_{\tilde{A}}\left(x_{i}\right)\right)}^{\lambda },{\left(\nu_{\tilde{A}}\left(x_{i}\right)\right)}^{\lambda }),\lambda >0$$ (6)

Definition 5: Let *E*(*A*) be

$$E\left(A\right)=\left\{\begin{array}{c} -\mu_{A}ln\mu_{A}-\nu_{A}ln\nu_{A}-\pi_{A}ln\pi_{A},\mu_{A}+\nu_{A}\in \left(0,1\right)\\ 0\Leftrightarrow A=\left[0,1\right]\cup \;\;\left[1,0\right] \end{array}\right.$$

where E (*A*) satisfies the property axiom of fuzzy entropy.

Definition 5: Let the exact function of fuzzy set *A* be:

$$H\left(A\right)=\mu_{A}\left(x\right)+\nu_{A}\left(x\right)$$ (7)

by using intuitionistic fuzzy operation rules, it can be obtained that

$$H\left(A\right)=(\left(\mu_{A}\left(x\right)+\mu_{A}\left(x\right)\cdot \pi_{A}\left(x\right)\right)+\left(\nu_{A}\left(x\right)+\nu_{A}\left(x\right)\cdot \pi_{A}\left(x\right)\right)$$ (8)

where *H*(*A*) ∈ [0,1].

$\tilde{A}$ and $\tilde{B}$ also follow the following principles:

$$H\left(\tilde{A}\right)=(\left(\mu_{\tilde{A}}\left(x_{i}\right)+\mu_{\tilde{A}}\left(x_{i}\right)\cdot \pi_{\tilde{A}}\left(x_{i}\right)\right)+\left(\nu_{\tilde{A}}\left(x_{i}\right)+\nu_{\tilde{A}}\left(x_{i}\right)\cdot \pi_{\tilde{A}}\left(x_{i}\right)\right)$$ (9)

$$H\left(\tilde{B}\right)=(\left(\mu_{\tilde{B}}\left(x_{i}\right)+\mu_{\tilde{B}}\left(x_{i}\right)\cdot \pi_{\tilde{B}}\left(x_{i}\right)\right)+\left(\nu_{\tilde{B}}\left(x_{i}\right)+\nu_{\tilde{B}}\left(x_{i}\right)\cdot \pi_{\tilde{B}}\left(x_{i}\right)\right)$$ (10)

if $H\left(\tilde{A}\right)<H\left(\tilde{B}\right)$, then $\tilde{A}<\tilde{B}$.

if $H\left(\tilde{A}\right)=H\left(\tilde{B}\right)$, then $\tilde{A}=\tilde{B}$.

Definition 6: Set the score function of fuzzy set *A* be:

$$S\left(A\right)=\mu_{A}\left(x\right)-\nu_{A}\left(x\right)$$ (11)

by using intuitionistic fuzzy operation rules, it can be obtained that

$$S\left(A\right)=(\left(\mu_{A}\left(x\right)+\mu_{A}\left(x\right)\cdot \pi_{A}\left(x\right)\right)-\left(\nu_{A}\left(x\right)+\nu_{A}\left(x\right)\cdot \pi_{A}\left(x\right)\right)$$ (12)

where *S*(*A*) ∈ [-1,1].

$\tilde{A}$ and $\tilde{B}$ also follow the following principles:

$$S\left(\tilde{A}\right)=(\left(\mu_{\tilde{A}}\left(x_{i}\right)+\mu_{\tilde{A}}\left(x_{i}\right)\cdot \pi_{\tilde{A}}\left(x_{i}\right)\right)-\left(\nu_{\tilde{A}}\left(x_{i}\right)+\nu_{\tilde{A}}\left(x_{i}\right)\cdot \pi_{\tilde{A}}\left(x_{i}\right)\right)$$ (13)

$$S\left(\tilde{B}\right)=(\left(\mu_{\tilde{B}}\left(x_{i}\right)+\mu_{\tilde{B}}\left(x_{i}\right)\cdot \pi_{\tilde{B}}\left(x_{i}\right)\right)-\left(\nu_{\tilde{B}}\left(x_{i}\right)+\nu_{\tilde{B}}\left(x_{i}\right)\cdot \pi_{\tilde{B}}\left(x_{i}\right)\right)$$ (14)

if $S\left(\tilde{A}\right)\geq S\left(\tilde{B}\right)$, then $\tilde{A}\geq \tilde{B}$, vice versa.

if $S\left(\tilde{A}\right)=S\left(\tilde{B}\right)$, then $\tilde{A}=\tilde{B}$.

### 3.2 EWIF-AHP evaluation model operating description

The model uses the form of questionnaire to organize experts to investigate and analyze the influencing factors of intelligent transformation and development of logistics firms.

In EWIF-AHP model, two-factor evaluation criteria refers to the analytic hierarchy process 9 degrees labeling method [42], and the intuitionistic fuzzy number corresponding table is shown in Table 1.

**Table 1:** Evaluation scale table.

evaluation level	intuitionistic fuzzy number
Factor *i* is extremely more important than factor *j*	(0.90, 0.10, 0.00)
Factor *i* is significantly more important than factor *j*	(0.80, 0.15, 0.05)
Factor *i* is more important than factor *j*	(0.70, 0.20, 0.10)
Factor *i* is slightly more important than factor *j*	(0.60, 0.25, 0.15)
Factor *i* is as important as factor *j*	(0.50, 0.30, 0.20)
Factor *j* is slightly more important than factor *i*	(0.40, 0.45, 0.15)
Factor *j* is more important than factor *i*	(0.30, 0.60, 0.10)
Factor *j* is significantly more important than factor *i*	(0.20, 0.75, 0.05)
Factor *j* is extremely more important than factor *i*	(0.10, 0.90, 0.00)

The EWIF-AHP model specific steps are as follows:

Step 1: define model evaluation indicators, and the indicators are shown in Table 2. The indicators in Table 2 will be transformed into the intuitionistic fuzzy numbers, and the data correspond to the decision fuzzy number matrix *A*, which the elements in *A* = (*A*_*ij*_)_*m*×*n*_ are expressed by the intuitionistic fuzzy numbers.
  Entropy value of the *j*th indicator in the decision-making fuzzy matrix *A* is expressed as:
$$E_{j}=\frac{1}{2mln3}\sum_{i=1}^{n}((\mu_{ij}\cdot ln\mu_{ij})+\left(\nu_{ij}\cdot ln\nu_{ij}\right)+\left(\pi_{ij}\cdot ln\pi_{ij}\right))$$ (15)
  where entropy weight of *j* is:
$$\omega_{j}=\frac{1-E_{j}}{\sum_{j=1}^{n}\left(1-E_{j}\right)}$$ (16)
  and *ω*_*j*_ ∈ [0,1].
Step 2: the decision matrix will be assigned with entropy weight by function (16), and the weighted matrix *R* with fuzzy preference will be obtained, and *R* = (*R*_*ij*_)_*m*×*n*_ = (*ω*_*j*_*A*_*ij*_)_*m*×*n*_. The change rule will be:
$$r=\omega^{\left(l\right)}A=\left(\omega_{1}^{\left(l\right)},\omega_{2}^{\left(l\right)},\ldots ,\omega_{m}^{\left(l\right)}\right)\left[\begin{array}{ccc} a_{11} & a_{12} & \begin{array}{cc} \ldots & a_{1n} \end{array}\\ a_{21} & a_{22} & \begin{array}{cc} \cdots & a_{2n} \end{array}\\ \begin{array}{c} \cdots \\ a_{m1} \end{array} & \begin{array}{c} \cdots \\ a_{m1} \end{array} & \begin{array}{c} \begin{array}{cc} \cdots & \cdots \end{array}\\ \begin{array}{cc} a_{21} & a_{mn} \end{array} \end{array} \end{array}\right]=\left(\overset{m}{\underset{i=1}{\sum }}\omega_{i}^{\left(l\right)}a_{i1},\overset{m}{\underset{i=1}{\sum }}\omega_{i}^{\left(l\right)}a_{i2},\ldots ,\overset{m}{\underset{i=1}{\sum }}\omega_{i}^{\left(l\right)}a_{in}\right)$$
  *i* = 1,2,…*m*, *j* = 1,2,…, *n*, where *l* is the series of index level in intuitionistic fuzzy analysis, and the change rule of fuzzy entropy weight of the *j*th indicator is as follows:
$$r_{j}=\overset{m}{\underset{i=1}{\sum }}\omega_{i}^{\left(l\right)}a_{ij}=\left(1-\overset{m}{\underset{i=1}{\prod }}{\left(1-\mu_{ij}\right)}^{\omega_{i}^{\left(l\right)}},\overset{m}{\underset{i=1}{\prod }}\nu_{ij}^{\omega_{i}^{\left(l\right)}}\right)$$
$$\mu_{j}=1-\prod_{i=1}^{m}{\left(1-\mu_{ij}\right)}^{\omega_{i}},\;\nu_{j}=\prod_{i=1}^{m}\nu_{ij}^{\omega_{i}}$$
Step 3: By using function (5) to assemble the weighted matrix *R*, According to function (1), a comprehensive evaluation that can obtain information described by intuitionistic fuzzy sets for the *ith* time. *A*_*i*_ = (*μ*_*j*_, *ν*_*j*_), where *i* = 1,2,…*m*.
Step 4: according to function (8) and function (12), the exact function *H*(*A*) and the score function *S*(*A*) of the comprehensive evaluation matrix *A* will be obtained respectively.
Step 5: sort the indicators according to the scores, analyze the output results, and then evaluate the impact of each index.

**Table 2:** Index system of influencing factors on logistics firms’ smart transformation.

primary indicator	secondary indicator
Logistics technology innovation ability analysis A_1_	Perception technology equipment investment degree A_11_
	Connecting technical equipment investment degree A_12_
	Mobile technology equipment investment degree A_13_
	Infrastructure upgrades level A_14_
Logistics big data sharing ability analysis A_2_	Application level of internal information platform A_21_
	Compatibility level of external system A_22_
	Data standardization degree A_23_
	Information interconnection investment A_24_
Logistics management upgrade ability analysis A_3_	High-skilled personnel input A_31_
	Shared resource balancing level A_32_
	The combination degree of industry, politics, education and research A_33_
Logistics decision-making transformation ability analysis A_4_	Logistics demand level A_41_
	Policy guidance promotion A_42_
	Innovative driving degree of new logistics mode A_43_

## 4 Case study

In order to deeply understand the characteristics of China’s logistics firms, the problems encountered in the intelligent transformation and upgrading and the upgrading direction selected. The logistics firms in the case study are divided into two main bodies. One is the logistics firms with medium scale and above, and the other is the small and medium-sized logistics firms. There is currently no international consensus on the definition of small and medium-sized logistics enterprises and medium scale and above enterprises, and the definition of these enterprises varies in different countries or development stages. Distinguishing these types of enterprises is reflected in their strength and scale, position in the logistics market, competitiveness, and social influence. According to the "2017 Classification Method for Large, Medium, Small, and Micro Enterprises in Statistics" issued by the National Bureau of Statistics, this method divides Chinese enterprises into four types based on the characteristics of different types of enterprises, including the number of employees and operating income. This article compromises the indicators of transportation and warehousing industries. Taking into account the impact of various factors such as COVID-19 during the research period, the enterprises with a staff size of less than 500 and operating income of less than 50 million are classified as small and medium-sized enterprises, while those with a staff size and operating income exceeding these numbers are classified as medium-sized and above enterprises. The development goals and concerns of each classification subject are different, and their corresponding transformation and upgrading paths are also different.

Aim at deeply investigating and evaluating the direction and influencing factors of intelligent transformation of the two main logistics firms, more than 110 enterprises have been visited and investigated, which are distributed in eastern China, including the more developed big cities in the east and the less developed small cities. Then, summarize the current situation, problems and solutions of logistics intelligent transformation. First, take the logistics firms of medium scale and above as the research subject to evaluate their transformation and upgrading development path. In order to evaluate the factors affecting the upgrading and transformation, EWIF-AHP method has been adopted to organize experts to score and compare the factors. Experts are divided into three groups, which are from universities, enterprises and scientific research institutes. Among them, enterprise experts are all from medium-sized and above logistics enterprises, and most of them are from middle and senior managers of large logistics enterprises. Each group has six experts.

Three groups of experts used Delphi method to obtain the evaluation scores of influencing factors. Due to the professionalism and authority of each expert in their field, the initial intuitionistic fuzzy number directly selects the arithmetic mean score given by the experts. Compared the two indicators with each other, and converted the comparison results into intuitionistic fuzzy numbers corresponding to Table 1. The intuitionistic fuzzy number matrix corresponding to the primary indicators and the secondary indicators were shown in Tables 3 to 7.

**Table 3:** Intuitionistic fuzzy matrix A.

A	A_1_	A_2_	A_3_	A_4_
A_1_	(0.50,0.30,0.20)	(0.40,0.45,0.15)	(0.70,0.20,0.10)	(0.60,0.25,0.15)
A_2_	(0.60,0.25,0.15)	(0.50,0.30,0.20)	(0.80,0.15,0.05)	(0.70,0.20,0.10)
A_3_	(0.30,0.60,0.10)	(0.20,0.75,0.05)	(0.50,0.30,0.20)	(0.60,0.25,0.15)
A_4_	(0.40,0.45,0.15)	(0.30,0.60,0.10)	(0.40,0.45,0.15)	(0.50,0.30,0.20)

**Table 4:** Intuitionistic fuzzy matrix of logistics technology innovation A_1_.

A_1_	A_11_	A_12_	A_13_	A_14_
A_11_	(0.50,0.30,0.20)	(0.40,0.45,0.15)	(0.30,0.60,0.10)	(0.60,0.25,0.15)
A_12_	(0.60,0.25,0.15)	(0.50,0.30,0.20)	(0.30,0.60,0.10)	(0.70,0.20,0.10)
A_13_	(0.70,0.20,0.10)	(0.60,0.25,0.15)	(0.50,0.30,0.20)	(0.80,0.15,0.05)
A_14_	(0.40,0.45,0.15)	(0.30,0.60,0.10)	(0.20,0.75,0.05)	(0.50,0.30,0.20)

**Table 5:** Intuitionistic fuzzy matrix of logistics big data sharing A_2_.

A_2_	A_21_	A_22_	A_23_	A_24_
A_21_	(0.50,0.30,0.20)	(0.60,0.25,0.15)	(0.40,0.45,0.15)	(0.30,0.60,0.10)
A_22_	(0.40,0.45,0.15)	(0.50,0.30,0.20)	(0.30,0.60,0.10)	(0.20,0.75,0.05)
A_23_	(0.60,0.25,0.15)	(0.70,0.20,0.10)	(0.50,0.30,0.20)	(0.40,0.45,0.15)
A_24_	(0.70,0.20,0.10)	(0.80,0.15,0.05)	(0.60,0.25,0.15)	(0.50,0.30,0.20)

**Table 6:** Intuitionistic fuzzy matrix of logistics management upgrading A_3_.

A_3_	A_31_	A_32_	A_33_
A_31_	(0.50,0.30,0.20)	(0.60,0.25,0.15)	(0.40,0.45,0.15)
A_32_	(0.40,0.45,0.15)	(0.50,0.30,0.20)	(0.30,0.60,0.10)
A_33_	(0.60,0.25,0.15)	(0.70,0.20,0.10)	(0.50,0.30,0.20)

**Table 7:** Intuitionistic fuzzy matrix of logistics decision-making transformation A_4_.

A_4_	A_41_	A_42_	A_43_
A_41_	(0.50,0.30,0.20)	(0.50,0.30,0.20)	(0.40,0.45,0.15)
A_42_	(0.50,0.30,0.20)	(0.50,0.30,0.20)	(0.40,0.45,0.15)
A_43_	(0.60,0.25,0.15)	(00.60,0.25,0.15)	(0.50,0.30,0.20)

The intuitionistic fuzzy decision matrixes have been assigned entropy weights, and the intuitionistic fuzzy judgment matrix R has been transformed. According to the primary indicator and secondary indicator, *R*, *R*_1_, *R*_2_, *R*_3_, *R*_4_ have been output step by step.


$$R=\left[\begin{array}{ccc} \left(0.15,0.76\right) & \left(0.12,0.82\right) & \begin{array}{cc} \left(0.28,0.65\right) & \left(0.20,0.71\right) \end{array}\\ \left(0.19,0.72\right) & \left(0.16,073\right) & \begin{array}{cc} \left(0.35,0.60\right) & \left(0.26,0.67\right) \end{array}\\ \begin{array}{c} \left(0.08,0.89\right)\\ \left(0.11,0.83\right) \end{array} & \begin{array}{c} \left(0.06,0.93\right)\\ \left(0.09,0.88\right) \end{array} & \begin{array}{cc} \begin{array}{c} \left(0.17,0.72\right)\\ \left(0.13,0.81\right) \end{array} & \begin{array}{c} \left(0.20,0.71\right)\\ \left(0.16,0.74\right) \end{array} \end{array} \end{array}\right]$$

$$R_{1}=\left[\begin{array}{ccc} \left(0.15,0.75\right) & \left(0.11,0.83\right) & \begin{array}{cc} \left(0.09,0.88\right) & \left(0.22,0.69\right) \end{array}\\ \left(0.20,0.72\right) & \left(0.15,076\right) & \begin{array}{cc} \left(0.09,0.88\right) & \left(0.28,0.65\right) \end{array}\\ \begin{array}{c} \left(0.25,0.68\right)\\ \left(0.11,0.83\right) \end{array} & \begin{array}{c} \left(0.19,0.73\right)\\ \left(0.08,0.89\right) \end{array} & \begin{array}{cc} \begin{array}{c} \left(0.17,0.73\right)\\ \left(0.06,0.93\right) \end{array} & \begin{array}{c} \left(0.35,0.60\right)\\ \left(0.17,0.72\right) \end{array} \end{array} \end{array}\right]$$

$$R_{2}=\left[\begin{array}{ccc} \left(0.15,0.75\right) & \left(0.22,0.68\right) & \begin{array}{cc} \left(0.11,0.83\right) & \left(0.09,0.88\right) \end{array}\\ \left(0.12,0.83\right) & \left(0.17,0.08\right) & \begin{array}{cc} \left(0.08,0.89\right) & \left(0.06,0.93\right) \end{array}\\ \begin{array}{c} \left(0.20,0.72\right)\\ \left(0.25,0.68\right) \end{array} & \begin{array}{c} \left(0.28,0.64\right)\\ \left(0.36,0.59\right) \end{array} & \begin{array}{cc} \begin{array}{c} \left(0.15,0.76\right)\\ \left(0.19,0.72\right) \end{array} & \begin{array}{c} \left(0.13,0.82\right)\\ \left(0.16,0.74\right) \end{array} \end{array} \end{array}\right]$$

$$R_{3}=\left[\begin{array}{cc} \left(0.20,0.68\right) & \begin{array}{cc} \left(0.27,0.62\right) & \left(0.15,0.77\right) \end{array}\\ \begin{array}{c} \left(0.15,0.77\right)\\ \left(0.26,0.64\right) \end{array} & \begin{array}{cc} \begin{array}{c} \left(0.21,0.66\right)\\ \left(0.34,0.57\right) \end{array} & \begin{array}{c} \left(0.11,0.85\right)\\ \left(0.20,0.67\right) \end{array} \end{array} \end{array}\right]$$

$$R_{4}=\left[\begin{array}{cc} \left(0.21,0.66\right) & \begin{array}{cc} \left(0.21,0.70\right) & \left(0.15,0.77\right) \end{array}\\ \begin{array}{c} \left(0.21,0.70\right)\\ \left(0.27,0.63\right) \end{array} & \begin{array}{cc} \begin{array}{c} \left(0.21,0.67\right)\\ \left(0.27,0.63\right) \end{array} & \begin{array}{c} \left(0.15,0.77\right)\\ \left(0.20,0.67\right) \end{array} \end{array} \end{array}\right]$$

Through the matrixes integrated into the functions, the function scores of the influence indicator were obtained, as shown in Table 8.

**Table 8:** Final results of indexes.

Indicator	Score	Indicator	Score
A_1_	0.2443	A_24_	0.2990
A_11_	0.0952	A_3_	-0.0015
A_12_	0.2174	A_31_	0.1832
A_13_	0.2989	A_32_	-0.0273
A_14_	-0.1652	A_33_	0.2841
A_2_	0.2989	A_4_	-0.0411
A_21_	0.0983	A_41_	0.1150
A_22_	-0.1606	A_42_	0.1150
A_23_	0.2449	A_43_	0.2618

According to the score relation of intuitionistic fuzzy set defined by Professor Atanosuowu, *S*(*A*) ∈ [-1,1], logistics meaning of positive and negative value is the degree of belonging and not belonging, and the negative value can also be understood not to belong to the membership function, which has a weak influence on the target layer. It can be seen from Table 8 that among the primary indicators, *S*(*A*) = (0.2443, 0.2989, −0.0015, −0.0411), logistics big data sharing is the most influential factor affecting logistics transformation, followed by logistics technology innovation. The impact of logistics management and logistics decision-making on transformation and development is relatively weak compared with the other two. Although the following two values are negative, it indicates that upgrading should not be chosen. However, through conducting research on enterprises, it was found that logistics management and decision-making capabilities still need to be upgraded and transformed. However, during the implementation process, these two factors belong to the strategic level update, forming a relatively complete set of processes from the enterprise’s business scope, operational processes, and personnel management, and have certain habits. The difficulty and speed of updating are more challenging compared to logistics technology and logistics data. Therefore, even if it is a negative number, for the long-term development of the enterprise, we still consider the transformation and upgrading in this area.

In the evaluation of logistics technology innovation, *S*(*A*_1_) = (0.0952,0.2174,0.2989,−0.1652), perception technology, connection technology and mobile technology all have an important impact on technological innovation, among which mobile equipment has the greatest impact; From the data, it can be seen that the score for infrastructure upgrading is relatively low. This does not mean that infrastructure upgrading should not be carried out, but rather because most large logistics enterprises have relatively complete automation and intelligent implementation, and at the beginning of plant construction, most will estimate the future volume of goods. Therefore, relatively advanced infrastructure has be chosen, and there will left the certain interface for the subsequent introduction of intelligent devices. In the evaluation of logistics big data sharing, *S*(*A*_2_) = (0.0983,−0.1606,0.2449,0.2990), application level of logistics internal information platform, degree of data standardization and investment in information interconnection have a certain impact on logistics firms intelligent transformation, and investment in information interconnection has the greatest impact. From the data, it can be seen that the most difficult point lies in cross platform data integration. Through visiting and researching, it is known that large enterprises have a good foundation in the collection, communication, and analysis of remote data within their own enterprises, and their development intelligence is constantly improving. However, there are still certain difficulties in implementing updates for data conversion and analysis between platforms, especially for data sharing between enterprises. In addition to having a certain degree of corporate privacy, there is also a certain relationship between companies, their level of closeness, and even changes in the financial market. Therefore, there are certain difficulties in the execution process. In upgrading logistics management, *S*(*A*_3_) = (0.1832,−0.0273,0.2841), high-skilled personnel input and the degree of combination of industry, government, university and research have a certain positive impact. From the data, it can be seen that shared resources have certain difficulties in updating and developing compared to other factors, especially for employee sharing. Large enterprises hardly use shared employees because they have a relatively complete personnel management system, formulate personnel management plans in accordance with laws and labor contracts, and each enterprise has its own special requirements and high authority. Therefore, in special periods, the principle of personnel sharing is adopted, After leaving the special period, there are indeed certain difficulties in executing the enterprise. In the transformation of logistics decision-making, *S*(*A*_4_) = (0.1150,0.1150,0.2618), the level of logistics demand, the promotion degree of logistics policy and the innovation driving degree of new logistics model all have a certain influence on intellectualization of logistics decision-making, among which the innovation driving degree of new logistics model is the largest.

For medium-sized and above logistics firms, their development and operation directions are mainly third-party logistics, with relatively perfect mechanism and certain intelligent technology, and their key development is data sharing. Big data sharing is not only to establish the seamless connection of the national transportation network platform, but also to realize the multi-point real-time synchronization mechanism in the process of data retrieval, visualization and fast tracking in the storage and transfer center. The biggest difference between medium-sized logistics firms and super large logistics enterprises lies in their types. Super large logistics enterprises are mainly state-owned holding enterprises and super large e-commerce enterprises supporting logistics, such as China Railway, China Post, China Cosoc Shipping etc. Most medium-sized logistics firms are private enterprises, many of which serve super large logistics enterprises, and some of them are even new enterprises independent of their subordinate enterprises. While serving large logistics enterprises, the establishment of their logistics network nodes is gradually improved. In order to coordinate with the rapid transportation and distribution capabilities of large logistics enterprises, the hardware and software in the field of technical intelligence of medium-sized logistics firms have been developed to a certain extent through more than 20 years of development. At the same time, in terms of logistics management and decision-making development, most of the time, large-scale logistics enterprises have been benchmarking their management models. For medium-sized logistics firms, they have a higher requirement standard and a more "wolf" enterprise concept of striving for development. Therefore, how to integrate the gradually expanding logistics nodes, how to face the rapid increase of data in the development from domestic to cross-border business, is an urgent problem faced by medium-sized and above logistics firms. In the process of stable development and intelligent reform, medium-sized and above logistics firms take big data sharing mechanism as the first choice to solve this problem.

The second analysis subject is small and medium-sized logistics firms. In terms of ownership, small and medium-sized logistics firms are basically private enterprises, and there are many small and medium-sized logistics firms developed in the Yangtze River Delta region in southeast China. The business scope of small and medium-sized logistics firms is mainly divided into two directions. One is to serve small regional logistics and undertake regional business outsourcing of large logistics enterprises. Most of the services are mainly distribution, simple processing, logistics customs declaration, freight forwarding and shipping agency services; The other part serves small and medium-sized manufacturing enterprises, many of which focus on production logistics. Some are equivalent to the fourth party logistics, providing part-time services; Some are separated by small and medium-sized manufacturing logistics, with strong production logistics operation background. Compared with large and medium-sized logistics firms, the development of small and medium-sized logistics firms is different from the current situation.

In order to discuss the influencing factors of the upgrading and transformation of small and medium-sized logistics firms in the context of smart logistics, experts were organized to score and compare the influencing factors. Experts were divided into three groups, respectively from universities, small and medium-sized enterprises, and large logistics enterprises. Among them, small and medium-sized logistics enterprise experts are the core managers of small and medium-sized logistics firms and small and medium-sized manufacturing enterprises. Since the logistics and manufacturing industry in the Yangtze River Delta region is a relatively developed region for China’s small and medium-sized logistics enterprises, two-thirds of the experts come from the Yangtze River Delta region, and the business status of the enterprises in the region is also highly universal.

Three groups of experts used Delphi method to obtain the evaluation scores of influencing factors. Compared the two indicators with each other, and converted the comparison results into intuitionistic fuzzy numbers corresponding to Table 1. The intuitionistic fuzzy number matrix corresponding to the primary indicators and the secondary indicators were shown in Tables 9 to 13.

**Table 9:** Intuitionistic fuzzy matrix A.

A	A_1_	A_2_	A_3_	A_4_
A_1_	(0.50,0.30,0.20)	(0.70,0.20,0.10)	(0.40,0.45,0.15)	(0.80,0.15,0.05)
A_2_	(0.30,0.60,0.10)	(0.50,0.30,0.20)	(0.30,0.60,0.0.1)	(0.60,0.25,0.15)
A_3_	(0.60,0.25,0.15)	(0.70,0.20,0.10)	(0.50,0.30,0.20)	(0.80,0.15,0.05)
A_4_	(0.20,0.75,0.05)	(0.40,0.45,0.15)	(0.20,0.75,0.05)	(0.50,0.30,0.20)

**Table 10:** Intuitionistic fuzzy matrix of logistics technology innovation A_1_.

A_1_	A_11_	A_12_	A_13_	A_14_
A_11_	(0.50,0.30,0.20)	(0.60,0.25,0.15)	(0.50,0.30,0.20)	(0.70,0.20,0.10)
A_12_	(0.40,0.45,0.15)	(0.50,0.30,0.20)	(0.40,0.45,0.15)	(0.60,0.25,0.15)
A_13_	(0.50,0.30,0.20)	(0.60,0.25,0.15)	(0.50,0.30,0.20)	(0.70,0.20,0.10)
A_14_	(0.30,0.60,0.10)	(0.40,0.45,0.15)	(0.30,0.60,0.10)	(0.50,0.30,0.20)

**Table 11:** Intuitionistic fuzzy matrix of logistics big data sharing A_2_.

A_2_	A_21_	A_22_	A_23_	A_24_
A_21_	(0.50,0.30,0.20)	(0.70,0.20,0.10)	(0.60,0.25,0.15)	(0.70,0.20,0.10)
A_22_	(0.30,0.60,0.10)	(0.50,0.30,0.20)	(0.40,0.45,0.15)	(0.50,0.30,0.20)
A_23_	(0.40,0.45,0.15)	(0.60,0.25,0.15)	(0.50,0.30,0.20)	(0.60,0.25,0.15)
A_24_	(0.30,0.60,0.10)	(0.50,0.30,0.20)	(0.40,0.45,0.15)	(0.50,0.30,0.20)

**Table 12:** Intuitionistic fuzzy matrix of logistics management upgrading A_3_.

A_3_	A_31_	A_32_	A_33_
A_31_	(0.50,0.30,0.20)	(0.80,0.15,0.05)	(0.70,0.20,0.10)
A_32_	(0.20,0.75,0.05)	(0.50,0.30,0.20)	(0.40,0.45,0.15)
A_33_	(0.30,0.60,0.10)	(0.60,0.25,0.15)	(0.50,0.30,0.20)

**Table 13:** Intuitionistic fuzzy matrix of logistics decision-making transformation A_4_.

A_4_	A_41_	A_42_	A_43_
A_41_	(0.50,0.30,0.20)	(0.60,0.25,0.15)	(0.70,0.20,0.10)
A_42_	(0.40,0.45,0.15)	(0.50,0.30,0.20)	(0.60,0.25,0.15)
A_43_	(0.30,0.60,0.10)	(0.40,0.45,0.15)	(0.50,0.30,0.20)

The intuitionistic fuzzy decision matrixes have been assigned entropy weights, and the intuitionistic fuzzy judgment matrix R has been transformed. According to the primary indicator and secondary indicator, *R*, *R*_1_, *R*_2_, *R*_3_, *R*_4_ have been output step by step.


$$R=\left[\begin{array}{ccc} \left(0.16,0.74\right) & \left(0.25,0.68\right) & \begin{array}{cc} \left(0.12,0.83\right) & \left(0.36,0.59\right) \end{array}\\ \left(0.08,0.88\right) & \left(0.15,0.75\right) & \begin{array}{cc} \left(0.08,0.88\right) & \left(0.22,0.68\right) \end{array}\\ \begin{array}{c} \left(0.20,0.71\right)\\ \left(0.05,0.93\right) \end{array} & \begin{array}{c} \left(0.25,0.68\right)\\ \left(0.11,0.83\right) \end{array} & \begin{array}{cc} \begin{array}{c} \left(0.15,0.75\right)\\ \left(0.05,0.93\right) \end{array} & \begin{array}{c} \left(0.36,0.59\right)\\ \left(0.17,0.72\right) \end{array} \end{array} \end{array}\right]$$

$$R_{1}=\left[\begin{array}{ccc} \left(0.15,0.75\right) & \left(0.20,0.71\right) & \begin{array}{cc} \left(0.15,0.75\right) & \left(0.28,0.64\right) \end{array}\\ \left(0.16,0.83\right) & \left(0.16,0.74\right) & \begin{array}{cc} \left(0.12,0.83\right) & \left(0.22,0.68\right) \end{array}\\ \begin{array}{c} \left(0.15,0.75\right)\\ \left(0.08,0.88\right) \end{array} & \begin{array}{c} \left(0.20,0.71\right)\\ \left(0.11,0.82\right) \end{array} & \begin{array}{cc} \begin{array}{c} \left(0.15,0.75\right)\\ \left(0.08,0.88\right) \end{array} & \begin{array}{c} \left(0.28,0.64\right)\\ \left(0.17,0.72\right) \end{array} \end{array} \end{array}\right]$$

$$R_{2}=\left[\begin{array}{ccc} \left(0.16,0.74\right) & \left(0.26,0.66\right) & \begin{array}{cc} \left(0.20,0.72\right) & \left(0.26,0.66\right) \end{array}\\ \left(0.09,0.88\right) & \left(0.16,0.74\right) & \begin{array}{cc} \left(0.11,0.83\right) & \left(0.16,0.74\right) \end{array}\\ \begin{array}{c} \left(0.12,0.82\right)\\ \left(0.09,0.88\right) \end{array} & \begin{array}{c} \left(0.21,0.70\right)\\ \left(0.16,0.76\right) \end{array} & \begin{array}{cc} \begin{array}{c} \left(0.15,0.76\right)\\ \left(0.11,0.83\right) \end{array} & \begin{array}{c} \left(0.21,0.70\right)\\ \left(0.16,0.74\right) \end{array} \end{array} \end{array}\right]$$

$$R_{3}=\left[\begin{array}{cc} \left(0.21,0.67\right) & \begin{array}{cc} \left(0.42,0.52\right) & \left(0.32,0.60\right) \end{array}\\ \begin{array}{c} \left(0.07,0.91\right)\\ \left(0.11,0.84\right) \end{array} & \begin{array}{cc} \begin{array}{c} \left(0.21,0.66\right)\\ \left(0.27,0.62\right) \end{array} & \begin{array}{c} \left(0.15,0.77\right)\\ \left(0.20,0.68\right) \end{array} \end{array} \end{array}\right]$$

$$R_{4}=\left[\begin{array}{cc} \left(0.20,0.67\right) & \begin{array}{cc} \left(0.26,0.64\right) & \left(0.34,0.57\right) \end{array}\\ \begin{array}{c} \left(0.15,0.77\right)\\ \left(0.11,0.85\right) \end{array} & \begin{array}{cc} \begin{array}{c} \left(0.20,0.68\right)\\ \left(0.15,0.77\right) \end{array} & \begin{array}{c} \left(0.27,0.62\right)\\ \left(0.21,0.66\right) \end{array} \end{array} \end{array}\right]$$

Through the matrixes integrated into the functions, the function scores of the influence indicator were obtained, as shown in Table 14.

**Table 14:** Final results of indexes.

Indicator	Score	Indicator	Score
A_1_	0.2822	A_24_	0.0435
A_11_	0.2732	A_3_	0.2991
A_12_	0.1441	A_31_	0.2990
A_13_	0.2732	A_32_	-0.1085
A_14_	-0.0936	A_33_	0.1291
A_2_	0.0516	A_4_	-0.2261
A_21_	0.2931	A_41_	0.2841
A_22_	0.0435	A_42_	0.1832
A_23_	0.2150	A_43_	-0.0273

According to Table 14, the biggest factor affecting the intelligent transformation of small and medium-sized logistics firms is the upgrading of logistics management, followed by the logistics technology upgrading, which is almost as important as the logistics management upgrading. Then, followed by logistics big data sharing and logistics decision-making transformation. In the upgrading of logistics management, with *S*(*A*_3_) = (0.2990, −0.1085, 0.1291), the input of highly skilled personnel and the degree of integration of industry, politics, education and research have a positive impact on the upgrading of logistics management. Through visiting and researching, it is known that the development of industry, politics, academia, and research poses certain difficulties for small and medium-sized enterprises compared to other factors. Small and medium-sized enterprises will only conduct industry academia research and development exhibitions after stabilizing their operating income and having a certain influence within their jurisdiction. One reason is that due to their own strengths, even if they conduct industry university research and development exhibitions, they still seek breakthroughs and transformations in logistics technology levels. The demand for strategic and tactical levels is not high, so are the cooperation funds. The second reason why enterprises choose industry university research cooperation is more to expand their influence, attract fresh graduates, and at the same time, provide certain government preferential policies. But this is a two-way choice. If the enterprise is small in scale, it is difficult to obtain industry university research projects. In the evaluation of logistics technology innovation, *S*(*A*_1_) = (0.2732,0.1441,0.2732,−0.0936). Perceptual technology, mobile technology, and connectivity technology all have an important impact on technology innovation, of which perceptual equipment has the greatest impact; Compared to other factors, the overall demand for infrastructure upgrades is relatively weak. For small and medium-sized enterprises, upgrading and renovating infrastructure requires a significant amount of funding. At the same time, after upgrading the infrastructure, the original equipment cannot be used or more advanced equipment needs to be introduced, so more self-investment is needed. After upgrading and renovating both infrastructure and equipment, it means an increase in operating costs and a significant improvement in logistics business processing capabilities. If the demand for logistics business in enterprises does not increase correspondingly, and there is no clear possibility of a significant increase in business volume in the future, it will cause certain waste for the enterprise. In the evaluation of logistics big data sharing, with S(A_2_) = (0.2931,0.0435,0.2150,0.0435), the application level of logistics internal information platform, the degree of data standardization, and the investment in information connectivity all have a certain impact on the intelligent transformation of logistics enterprises, and the application level of logistics internal information platform has the largest impact. Logistics decision-making transformation has the least negative impact on the intelligent transformation and upgrading of small and medium-sized logistics firms. In the logistics decision-making transformation, with *S*(*A*_4_) = (0.2841,0.1832,−0.0273), the level of logistics demand and the degree of promotion of logistics policies have a certain influence on the intelligence of logistics decision-making. Compared with the logistics transformation of small and medium-sized firms, the innovation driven degree of the new logistics model is far away. Because this requires strong market demand and high research capabilities, as well as significant capital investment, usually the amount invested is not proportional to the output. For small and medium-sized enterprises, the implementation difficulty of this factor is relatively high, and its adaptability is not strong.

**Fig 2:**
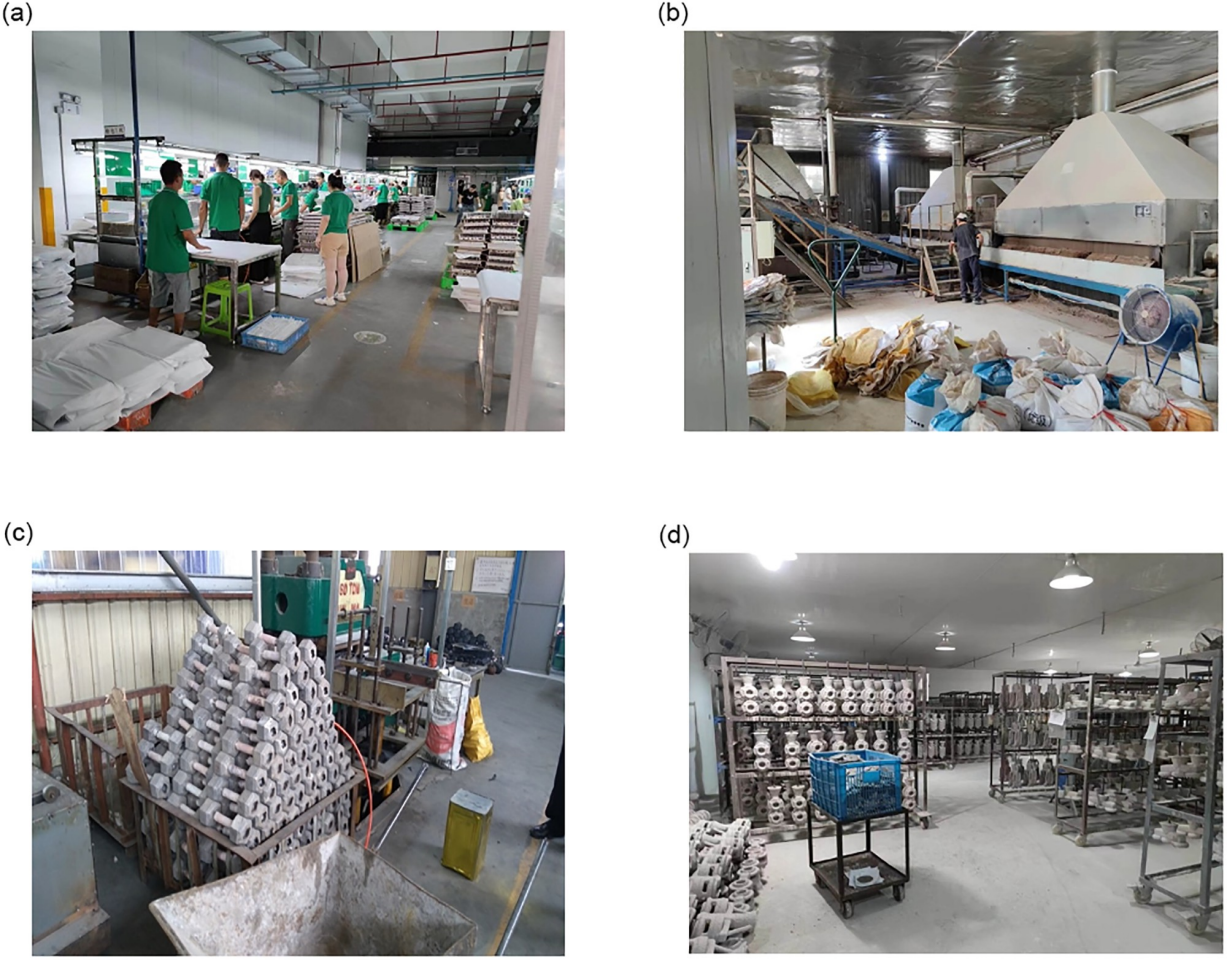
Logistics site environment of small and medium-sized logistics firms.

At this stage, the biggest dilemma faced by small and medium-sized logistics firms is the backwardness of management means and technology. Through investigation and interview, it has been found that most of the operators, managers and operators of small and medium-sized logistics firms in the Yangtze River Delta have college degree or below, and have little contact with new technologies and new management methods, so the overall degree of automation was low. Take production logistics and warehousing as an example. Most operations are manual operations. In addition, the operation process of warehousing inventory and order processing is mostly recorded manually, and the cycle time is in months and quarters, with a long-time interval. The development of small and medium-sized logistics firms focuses on cost and output, followed by the advanced intelligence of logistics. Simultaneously, for the on-site logistics of many manufacturing enterprises, the operation location and connection method did not apply the scientific layout principle, but were placed randomly according to the order of machines entering the site. As a result of this arrangement, the logistics efficiency was low, the staff surplus was excessive, and the logistics operation had potential safety hazards, as shown in Fig 2. Fig 2 shows the site layout problems encountered by the visited small and medium-sized logistics firms. Most of them have common problems such as large number of people, clutter, high manual work, single equipment, low utilization rate, and potential safety hazards in material handling. It can be seen that in small and medium-sized logistics firms, the key task of their transformation is to disassemble each link of logistics, extract each key operation point, analyze and evaluate each key link and process one by one, evaluate their logistics strength and link connection strength, summarize the root causes of problems, and solve the problems by means of management. The smart transformation and upgrading of logistics of small and medium-sized firms usually start with on-site improvement and make breakthroughs one by one to achieve overall progress.

In solving the problems of links and processes, the complexity and productivity of the operation links should also be considered. For simple, highly repetitive and intensive processes, new logistics technologies can be replaced, and automated machines can be used to replace manual processes. However, due to the low degree of intelligence and weak intelligence foundation of small and medium-sized logistics firms, they cannot undertake the fully automated production processes like those of large logistics enterprises. At the same time, most of the employees in enterprises have relatively low educational background, and their own skills and knowledge reserves cannot support intelligent fully automated process operations. Compared with the employment cost of new technical personnel, firms cannot achieve a large number of high-level personnel employments. In the meantime, large intelligent logistics equipments are mostly non-open source programs, and it is difficult for employees and engineers of small and medium-sized logistics firms to debug, upgrade and redevelop them. Therefore, in the application of new logistics technologies, perception technology and mobile technology are the core methods of the development of small and medium-sized firms. As mentioned above, on the one hand, this technology is generally low in price, convenient in operation and fast in manual operation; On the other hand, these technologies are necessary for the current industry. Additionally, mobile technology and sensing technology are mostly small devices, with high cost and practical feasibility. Therefore, small and medium-sized logistics firms must carry out forced transformation on simple intelligent technologies to meet market demand.

For the construction of logistics sharing platform, small and medium-sized logistics firms are gradually promoting the software update. Taking the warehousing system as an example, its data sharing platform updates fastest. At the same time, production logistics is also gradually promoting Kanban management. For the logistics of small and medium-sized firms, the data sharing mostly stays in the internal data, and the external data sharing cannot be realized at present. First, small and medium-sized logistics firms do not have too much work volume to cooperate with external logistics enterprises; Second, even if small and medium-sized logistics firms need to establish an external big data sharing platform, most of them are directly shared by external enterprise data platforms. Some small enterprises plan to directly replace the existing platform with the data platform of large enterprises; Some request professional data platform companies to help them develop data interfaces so that they can seamlessly interface with big data platforms. At present, small and medium-sized firms seldom consider the influencing factors of logistics decision-making transformation. The reason is that the current level of development is not enough to discuss the transformation of new models. When discussing the transformation, the experts and enterprises mentioned above mostly think that the transformation of small and medium-sized logistics firms is to give up logistics and completely transform into other industries.

## 5 Suggestion and conclusion

Logistics industry is a basic, strategic and forward-looking industry supporting development of national economy. Its intelligent and intelligent development degree directly affects the sustainability and efficiency of China’s primary, secondary and tertiary industries. Intelligent transformation of logistics industry should take logistics firms as the main bodies, and promote intelligent transformation of the whole industry from the micro level of intelligent development. Therefore, according to the different scale of logistics enterprises, the following different countermeasures are proposed.

For large and medium-sized logistics firms, the main countermeasures for their intelligent logistics transformation are as follows:

a. Strengthen technological innovation. Technology is the core element of the lasting and long-term development of firms, industries and industries. Technological innovation enables firms to survive and develop in market competition. According to the evaluation data, logistics firms should focus on the development of logistics connection technology, logistics mobile technology and logistics perception technology. The introduction of advanced logistics equipment and the innovation of logistics technology directly affect the operation volume and personnel employment ratio of firms per unit time. At the same time, it is also an effective way to break through the cost reduction and efficiency increase.
b. Strengthen the construction of digital sharing. Data sharing is a prerequisite for intelligence. Data sharing has gradually formed a big data exchange center to establish multiple communication channels for firms internally and externally. In addition, cloud technology, multi networking center and mobile terminal investment are the main concerns of firms in infrastructure investment. The advanced nature of infrastructure investment directly affects the sharing and analysis ability of data in the later stage.
c. Strengthen the construction of talent team and combination degree of industry, politics and science. In the transformation of China’s logistics from traditional mode to intelligent mode, in addition to the investment of hardware conditions such as technology and equipment, talent construction and knowledge sharing are the core soft powers. The construction of firm’s logistics talents needs to be carried out simultaneously with the transformation of talent cultivation and the introduction of high-level talents to avoid the lack of capacity caused by the brain losing. At the same time, the strong cooperation of industries, governments and researches will bring advanced development concepts and practical solutions to firm’s management tools, management technology, strategic direction and technological development, and also inject vitality and motivation into the overall smart transformation.

For small and medium-sized logistics firms, the main countermeasures for their intelligent logistics transformation are as follows:

a) Strengthen the upgrading of logistics management. The upgrading of logistics management is the primary problem that small and medium-sized logistics firms face in the transformation. In order to solve the logistics transformation, firms need to disassemble the processes and operations related to logistics and manufacturing, analyze their strength and rationality one by one, and determine which processes are unreasonable, which management is relatively backward, and which links need to be solved urgently. Therefore, we will attack and solve the problem point by point.
b) Strengthen logistics technology upgrading. In the logistics technology upgrading, small and medium-sized firms are quite different from large and medium-sized enterprises. Large and medium-sized firms have relatively advanced logistics technology, and have adopted some automated logistics parts, equipment, etc. However, small firms mainly rely on manpower, and increase their applications of simple sensing technology and mobile technology to meet the market demand. For large-scale intelligent logistics equipment, it is only suitable for manual replacement in operation links with high labor intensity, high repeatability and simple process. It is impossible to realize the intelligent construction of all regions, because its infrastructure and staffing cannot match the large-scale renewal. Therefore, small and medium-sized logistics firms can start with independent logistics equipment and simple and cheap logistics parts.
c) Strengthen internal data platform sharing of enterprises. In order to improve their production efficiency and operation efficiency, small and medium-sized logistics firms need to digitize and informationize their internal function modules. The new hardware, such as software and logistics perception technology, is used to improve the speed of information reading, computing and other operations. Meanwhile, the sharing of the internal data platform of the enterprise is conducive to the tracking of its development process, and is conducive to making more scientific decisions. Enterprise internal informatization also provides a basic docking platform for the later development of external networking informatization.

## Supporting information

S1 File The specific intuitionistic fuzzy calculation process of two kinds of logistics companies. (DOCX)
