# Supplementary material for: Influencing factors and countermeasures on intelligent transformation and upgrading of logistics firms: A case study in China
Source: PLoS One. 2024 Apr 4;19(4):e0297663. doi: 10.1371/journal.pone.0297663 (PMC10994377; doi:10.1371/journal.pone.0297663)
Supplement: S1 File — (DOCX) [file pone.0297663.s001.docx]

**Supporting Information**

**For logistics firms above medium scale.**

The intuitionistic fuzzy matrix could be shown in table.

**Table 3 Intuitionistic fuzzy matrix A**

| A | A_1_ | A_2_ | A_3_ | A_4_ | |
| --- | --- | --- | --- | --- | --- |
| A_1_ | (0.50,0.30,0.20) | (0.40,0.45,0.15) | (0.70,0.20,0.10) | | (0.60,0.25,0.15) |
| A_2_ | (0.60,0.25,0.15) | (0.50,0.30,0.20) | (0.80,0.15,0.05) | | (0.70,0.20,0.10) |
| A_3_ | (0.30,0.60,0.10) | (0.20,0.75,0.05) | (0.50,0.30,0.20) | | (0.60,0.25,0.15) |
| A_4_ | (0.40,0.45,0.15) | (0.30,0.60,0.10) | (0.40,0.45,0.15) | | (0.50,0.30,0.20) |

The entropy values could be $E_{A}= (0.5880, 0.5500, 0.5241, 0.5623)$.

The entropy weights could be $\omega_{A}=(0.2321,0.2534 ,0.2680 ,0.2465)$.

The intuitionistic fuzzy decision matrixes have been assigned entropy weights, and the intuitionistic fuzzy judgment matrix R has been transformed.

$$R=\left[ \begin{matrix} (0.15,0.76) & (0.12,0.82) & \begin{matrix} (0.28,0.65) & (0.20,0.71) \end{matrix} \\ (0.19,0.72) & (0.16,073) & \begin{matrix} (0.35,0.60) & (0.26,0.67) \end{matrix} \\ \begin{matrix} (0.08,0.89) \\ (0.11,0.83) \end{matrix} & \begin{matrix} (0.06,0.93) \\ (0.09,0.88) \end{matrix} & \begin{matrix} \begin{matrix} (0.17,0.72) \\ (0.13,0.81) \end{matrix} & \begin{matrix} (0.20,0.71) \\ (0.16,0.74) \end{matrix} \end{matrix} \end{matrix} \right]$$

Because the experts have divided into three groups, $R$ will be assembled three times according to function (5), with the comprehensive evaluation values being $A^{1}=(0.5678,0.2851)$, $A^{2}=\left( 0.6726,0.2661 \right)$, $A^{3}=\left( 0.4236,0.4249 \right)$

The score function $S\left( A \right)=(0.2443, 0.2989,-0.0015, -0.0411)$.

The intuitionistic fuzzy matrix could be shown in table.

**Table 4 Intuitionistic fuzzy matrix of logistics technology innovation A_1_**

| A_1_ | A_11_ | A_12_ | A_13_ | A_14_ | |
| --- | --- | --- | --- | --- | --- |
| A_11_ | (0.50,0.30,0.20) | (0.40,0.45,0.15) | (0.30,0.60,0.10) | | (0.60,0.25,0.15) |
| A_12_ | (0.60,0.25,0.15) | (0.50,0.30,0.20) | (0.30,0.60,0.10) | | (0.70,0.20,0.10) |
| A_13_ | (0.70,0.20,0.10) | (0.60,0.25,0.15) | (0.50,0.30,0.20) | | (0.80,0.15,0.05) |
| A_14_ | (0.40,0.45,0.15) | (0.30,0.60,0.10) | (0.20,0.75,0.05) | | (0.50,0.30,0.20) |

The entropy values could be $E_{A_{1}}= ( 0.5734, 0.5880, 0.5329, 0.5131)$.

The entropy weights could be $\omega_{A_{1}}=(0.2380 , 0.2298, 0.2605, 0.2716)$.

The intuitionistic fuzzy decision matrixes have been assigned entropy weights, and the intuitionistic fuzzy judgment matrix $R_{1}$ has been transformed.

$$R_{1}=\left[ \begin{matrix} (0.15,0.75) & (0.11,0.83) & \begin{matrix} (0.09,0.88) & (0.22,0.69) \end{matrix} \\ (0.20,0.72) & (0.15,076) & \begin{matrix} (0.09,0.88) & (0.28,0.65) \end{matrix} \\ \begin{matrix} (0.25,0.68) \\ (0.11,0.83) \end{matrix} & \begin{matrix} (0.19,0.73) \\ (0.08,0.89) \end{matrix} & \begin{matrix} \begin{matrix} (0.17,0.73) \\ (0.06,0.93) \end{matrix} & \begin{matrix} (0.35,0.60) \\ (0.17,0.72) \end{matrix} \end{matrix} \end{matrix} \right]$$

The comprehensive evaluation values being $A_{1}^{1}=(0.4643, 0.3754)$, $A_{1}^{2}=\left( 0.5495, 0.3082 \right)$, $A_{1}^{3}=\left( 0.6720, 0.2164 \right)$

The score function $S\left( A_{1} \right)=(0.0952,0.2174,0.2989,-0.1652)$.

The intuitionistic fuzzy matrix could be shown in table.

**Table 5 Intuitionistic fuzzy matrix of logistics big data sharing A_2_**

| A_2_ | A_21_ | A_22_ | A_23_ | A_24_ | |
| --- | --- | --- | --- | --- | --- |
| A_21_ | (0.50,0.30,0.20) | (0.60,0.25,0.15) | (0.40,0.45,0.15) | | (0.30,0.60,0.10) |
| A_22_ | (0.40,0.45,0.15) | (0.50,0.30,0.20) | (0.30,0.60,0.10) | | (0.20,0.75,0.05) |
| A_23_ | (0.60,0.25,0.15) | (0.70,0.20,0.10) | (0.50,0.30,0.20) | | (0.40,0.45,0.15) |
| A_24_ | (0.70,0.20,0.10) | (0.80,0.15,0.05) | (0.60,0.25,0.15) | | (0.50,0.30,0.20) |

The entropy values could be $E_{A_{2}}= ( 0.5734 , 0.5131, 0.5880, 0.5500)$.

The entropy weights could be $\omega_{A_{2}}=(0.2403, 0.2742, 0.2321, 0.2534)$.

The intuitionistic fuzzy decision matrixes have been assigned entropy weights, and the intuitionistic fuzzy judgment matrix $R_{2}$ has been transformed.

$$R_{2}=\left[ \begin{matrix} (0.15,0.75) & (0.22,0.68) & \begin{matrix} (0.11,0.83) & (0.09,0.88) \end{matrix} \\ (0.12,0.83) & (0.17,0.08) & \begin{matrix} (0.08,0.89) & (0.06,0.93) \end{matrix} \\ \begin{matrix} (0.20,0.72) \\ (0.25,0.68) \end{matrix} & \begin{matrix} (0.28,0.64) \\ (0.36,0.59) \end{matrix} & \begin{matrix} \begin{matrix} (0.15,0.76) \\ (0.19,0.72) \end{matrix} & \begin{matrix} (0.13,0.82) \\ (0.16,0.74) \end{matrix} \end{matrix} \end{matrix} \right]$$

The comprehensive evaluation values being $A_{2}^{1}=(0.4657, 0.3737)$, $A_{2}^{2}=\left( 0.3637, 0.4899 \right)$, $A_{2}^{3}=\left( 0.5686, 0.2847 \right)$

The score function $S\left( A_{2} \right)=(0.0983,-0.1606,0.2449,0.2990)$.

The intuitionistic fuzzy matrix could be shown in table.

**Table 6 Intuitionistic fuzzy matrix of logistics management upgrading A_3_**

| A_3_ | A_31_ | A_32_ | A_33_ |  |
| --- | --- | --- | --- | --- |
| A_31_ | (0.50,0.30,0.20) | (0.60,0.25,0.15) | (0.40,0.45,0.15) | |
| A_32_ | (0.40,0.45,0.15) | (0.50,0.30,0.20) | (0.30,0.60,0.10) | |
| A_33_ | (0.60,0.25,0.15) | (0.70,0.20,0.10) | (0.50,0.30,0.20) | |

The entropy values could be $E_{A_{3}}= ( 0.4517, 0.4201, 0.4457)$.

The entropy weights could be $\omega_{A_{3}}=(0.3259, 0.3447, 0.3294)$.

The intuitionistic fuzzy decision matrixes have been assigned entropy weights, and the intuitionistic fuzzy judgment matrix $R_{3}$ has been transformed.

$$R_{3}=\left[ \begin{matrix} (0.20,0.68) & \begin{matrix} (0.27,0.62) & (0.15,0.77) \end{matrix} \\ \begin{matrix} (0.15,0.77) \\ (0.26,0.64) \end{matrix} & \begin{matrix} \begin{matrix} (0.21,0.66) \\ (0.34,0.57) \end{matrix} & \begin{matrix} (0.11,0.85) \\ (0.20,0.67) \end{matrix} \end{matrix} \end{matrix} \right]$$

The comprehensive evaluation values being $A_{3}^{1}=(0.5084 ,0.3220)$, $A_{3}^{2}=\left( 0.4072, 0.4302 \right)$, $A_{3}^{3}=\left( 0.6101, 0.2458 \right)$

The score function $S\left( A_{3} \right)=(0.1832,-0.0273,0.2841)$.

The intuitionistic fuzzy matrix could be shown in table.

**Table 7 Intuitionistic fuzzy matrix of logistics decision-making transformation A_4_**

| A_4_ | A_41_ | A_42_ | A_43_ |  |
| --- | --- | --- | --- | --- |
| A_41_ | (0.50,0.30,0.20) | (0.50,0.30,0.20) | (0.40,0.45,0.15) | |
| A_42_ | (0.50,0.30,0.20) | (0.50,0.30,0.20) | (0.40,0.45,0.15) | |
| A_43_ | (0.60,0.25,0.15) | (00.60,0.25,0.15) | (0.50,0.30,0.20) | |

The entropy values could be $E_{A_{4}}= ( 0.449, 0.4499, 0.4628)$.

The entropy weights could be $\omega_{A_{4}}=(0.3359, 0.3359, 0.3281)$.

The intuitionistic fuzzy decision matrixes have been assigned entropy weights, and the intuitionistic fuzzy judgment matrix $R_{4}$ has been transformed.

$$R_{4}=\left[ \begin{matrix} (0.21,0.66) & \begin{matrix} (0.21,0.70) & (0.15,0.77) \end{matrix} \\ \begin{matrix} (0.21,0.70) \\ (0.27,0.63) \end{matrix} & \begin{matrix} \begin{matrix} (0.21,0.67) \\ (0.27,0.63) \end{matrix} & \begin{matrix} (0.15,0.77) \\ (0.20,0.67) \end{matrix} \end{matrix} \end{matrix} \right]$$

The comprehensive evaluation values being $A_{4}^{1}=(0.4692, 0.3609)$, $A_{4}^{2}=\left( 0.4692, 0.3609 \right)$, $A_{4}^{3}=\left( 0.5696, 0.2654 \right)$

The score function $S\left( A_{4} \right)=(0.1150,0.1150,0.2618)$.

**For logistics firms small and medium-sized scale.**

The intuitionistic fuzzy matrix could be shown in table.

**Table 9 Intuitionistic fuzzy matrix A**

| A | A_1_ | A_2_ | A_3_ | A_4_ | |
| --- | --- | --- | --- | --- | --- |
| A_1_ | (0.50,0.30,0.20) | (0.70,0.20,0.10) | (0.40,0.45,0.15) | | (0.80,0.15,0.05) |
| A_2_ | (0.30,0.60,0.10) | (0.50,0.30,0.20) | (0.30,0.60,0.0.1) | | (0.60,0.25,0.15) |
| A_3_ | (0.60,0.25,0.15) | (0.70,0.20,0.10) | (0.50,0.30,0.20) | | (0.80,0.15,0.05) |
| A_4_ | (0.20,0.75,0.05) | (0.40,0.45,0.15) | (0.20,0.75,0.05) | | (0.50,0.30,0.20) |

The entropy values could be $E_{A}= (0.5390, 0.5528, 0.5500, 0.4844)$.

The entropy weights could be $\omega_{A}=(0.2460, 0.2387, 0.2401, 0.2752)$.

The intuitionistic fuzzy decision matrixes have been assigned entropy weights, and the intuitionistic fuzzy judgment matrix R has been transformed.

$$R=\left[ \begin{matrix} (0.16,0.74) & (0.25,0.68) & \begin{matrix} (0.12,0.83) & (0.36,0.59) \end{matrix} \\ (0.08,0.88) & (0.15,0.75) & \begin{matrix} (0.08,0.88) & (0.22,0.68) \end{matrix} \\ \begin{matrix} (0.20,0.71) \\ (0.05,0.93) \end{matrix} & \begin{matrix} (0.25,0.68) \\ (0.11,0.83) \end{matrix} & \begin{matrix} \begin{matrix} (0.15,0.75) \\ (0.05,0.93) \end{matrix} & \begin{matrix} (0.36,0.59) \\ (0.17,0.72) \end{matrix} \end{matrix} \end{matrix} \right]$$

Because the experts have divided into three groups, $R$ will be assembled three times according to function (5), with comprehensive evaluation values being $A^{1}=(0.6406, 0.2481)$, $A^{2}=\left( 0.4462, 0.3997 \right)$, $A^{3}=\left( 0.6744, 0.2152 \right)$

The score function $S\left( A \right)=(0.2822, 0.0516, 0.2991, -0.2261)$.

The intuitionistic fuzzy matrix could be shown in table.

**Table 10 Intuitionistic fuzzy matrix of logistics technology innovation A_1_**

| A_1_ | A_11_ | A_12_ | A_13_ | A_14_ | |
| --- | --- | --- | --- | --- | --- |
| A_11_ | (0.50,0.30,0.20) | (0.60,0.25,0.15) | (0.50,0.30,0.20) | | (0.70,0.20,0.10) |
| A_12_ | (0.40,0.45,0.15) | (0.50,0.30,0.20) | (0.40,0.45,0.15) | | (0.60,0.25,0.15) |
| A_13_ | (0.50,0.30,0.20) | (0.60,0.25,0.15) | (0.50,0.30,0.20) | | (0.70,0.20,0.10) |
| A_14_ | (0.30,0.60,0.10) | (0.40,0.45,0.15) | (0.30,0.60,0.10) | | (0.50,0.30,0.20) |

The entropy values could be $E_{A_{1}}= ( 0.6019, 0.5940, 0.6019, 0.5417)$.

The entropy weights could be $\omega_{A_{1}}=(0.2397, 0.2445, 0.2397, 0.2760)$.

The intuitionistic fuzzy decision matrixes have been assigned entropy weights, and the intuitionistic fuzzy judgment matrix $R_{1}$ has been transformed.

$$R_{1}=\left[ \begin{matrix} (0.15,0.75) & (0.20,0.71) & \begin{matrix} (0.15,0.75) & (0.28,0.64) \end{matrix} \\ (0.16,0.83) & (0.16,0.74) & \begin{matrix} (0.12,0.83) & (0.22,0.68) \end{matrix} \\ \begin{matrix} (0.15,0.75) \\ (0.08,0.88) \end{matrix} & \begin{matrix} (0.20,0.71) \\ (0.11,0.82) \end{matrix} & \begin{matrix} \begin{matrix} (0.15,0.75) \\ (0.08,0.88) \end{matrix} & \begin{matrix} (0.28,0.64) \\ (0.17,0.72) \end{matrix} \end{matrix} \end{matrix} \right]$$

The comprehensive evaluation values being $A_{1}^{1}=(0.5888, 0.2565)$, $A_{1}^{2}=\left( 0.4869, 0.3465 \right)$, $A_{1}^{3}=\left( 0.5888, 0.2565 \right)$

The score function $S\left( A_{1} \right)=(0.2732,0.1441,0.2732,-0.0936)$.

The intuitionistic fuzzy matrix could be shown in table.

**Table 11 Intuitionistic fuzzy matrix of logistics big data sharing A_2_**

| A_2_ | A_21_ | A_22_ | A_23_ | A_24_ | |
| --- | --- | --- | --- | --- | --- |
| A_21_ | (0.50,0.30,0.20) | (0.70,0.20,0.10) | (0.60,0.25,0.15) | | (0.70,0.20,0.10) |
| A_22_ | (0.30,0.60,0.10) | (0.50,0.30,0.20) | (0.40,0.45,0.15) | | (0.50,0.30,0.20) |
| A_23_ | (0.40,0.45,0.15) | (0.60,0.25,0.15) | (0.50,0.30,0.20) | | (0.60,0.25,0.15) |
| A_24_ | (0.30,0.60,0.10) | (0.50,0.30,0.20) | (0.40,0.45,0.15) | | (0.50,0.30,0.20) |

The entropy values could be $E_{A_{2}}= ( 0.5819, 0.5763, 0.6050, 0.5763)$.

The entropy weights could be $\omega_{A_{2}}=(0.2518, 0.2552, 0.2379, 0.2552)$.

The intuitionistic fuzzy decision matrixes have been assigned entropy weights, and the intuitionistic fuzzy judgment matrix $R_{2}$ has been transformed.

$$R_{2}=\left[ \begin{matrix} (0.16,0.74) & (0.26,0.66) & \begin{matrix} (0.20,0.72) & (0.26,0.66) \end{matrix} \\ (0.09,0.88) & (0.16,0.74) & \begin{matrix} (0.11,0.83) & (0.16,0.74) \end{matrix} \\ \begin{matrix} (0.12,0.82) \\ (0.09,0.88) \end{matrix} & \begin{matrix} (0.21,0.70) \\ (0.16,0.76) \end{matrix} & \begin{matrix} \begin{matrix} (0.15,0.76) \\ (0.11,0.83) \end{matrix} & \begin{matrix} (0.21,0.70) \\ (0.16,0.74) \end{matrix} \end{matrix} \end{matrix} \right]$$

The comprehensive evaluation values being $A_{2}^{1}=(0.6347, 0.2336)$, $A_{2}^{2}=\left( 0.4317, 0.3934 \right)$, $A_{2}^{3}=\left( 0.5329, 0.3027 \right)$

The score function $S\left( A_{2} \right)=(0.2931,0.0435,0.2150,0.0435)$.

The intuitionistic fuzzy matrix could be shown in table.

**Table 12 Intuitionistic fuzzy matrix of logistics management upgrading A_3_**

| A_3_ | A_31_ | A_32_ | A_33_ |  |
| --- | --- | --- | --- | --- |
| A_31_ | (0.50,0.30,0.20) | (0.80,0.15,0.05) | (0.70,0.20,0.10) | |
| A_32_ | (0.20,0.75,0.05) | (0.50,0.30,0.20) | (0.40,0.45,0.15) | |
| A_33_ | (0.30,0.60,0.10) | (0.60,0.25,0.15) | (0.50,0.30,0.20) | |

The entropy values could be $E_{A_{3}}= ( 0.3967, 0.3914, 0.4311)$.

The entropy weights could be $\omega_{A_{3}}=(0.3388, 0.3418, 0.3195)$.

The intuitionistic fuzzy decision matrixes have been assigned entropy weights, and the intuitionistic fuzzy judgment matrix $R_{3}$ has been transformed.

$$R_{3}=\left[ \begin{matrix} (0.21,0.67) & \begin{matrix} (0.42,0.52) & (0.32,0.60) \end{matrix} \\ \begin{matrix} (0.07,0.91) \\ (0.11,0.84) \end{matrix} & \begin{matrix} \begin{matrix} (0.21,0.66) \\ (0.27,0.62) \end{matrix} & \begin{matrix} (0.15,0.77) \\ (0.20,0.68) \end{matrix} \end{matrix} \end{matrix} \right]$$

The comprehensive evaluation values being $A_{3}^{1}=(0.6895, 0.2080)$, $A_{3}^{2}=\left( 0.3785, 0.4658 \right)$, $A_{3}^{3}=\left( 0.4808, 0.3565 \right)$

The score function $S\left( A_{3} \right)=(0.2990,-0.1085,0.1291)$.

The intuitionistic fuzzy matrix could be shown in table.

**Table 13 Intuitionistic fuzzy matrix of logistics decision-making transformation A_4_**

| A_4_ | A_41_ | A_42_ | A_43_ |  |
| --- | --- | --- | --- | --- |
| A_41_ | (0.50,0.30,0.20) | (0.60,0.25,0.15) | (0.70,0.20,0.10) | |
| A_42_ | (0.40,0.45,0.15) | (0.50,0.30,0.20) | (0.60,0.25,0.15) | |
| A_43_ | (0.30,0.60,0.10) | (0.40,0.45,0.15) | (0.50,0.30,0.20) | |

The entropy values could be $E_{A_{4}}= ( 0.4457, 0.4517, 0.4201)$.

The entropy weights could be $\omega_{A_{4}}=(0.3294, 0.3259, 0.3447)$.

The intuitionistic fuzzy decision matrixes have been assigned entropy weights, and the intuitionistic fuzzy judgment matrix $R_{4}$ has been transformed.

$$R_{4}=\left[ \begin{matrix} (0.20,0.67) & \begin{matrix} (0.26,0.64) & (0.34,0.57) \end{matrix} \\ \begin{matrix} (0.15,0.77) \\ (0.11,0.85) \end{matrix} & \begin{matrix} \begin{matrix} (0.20,0.68) \\ (0.15,0.77) \end{matrix} & \begin{matrix} (0.27,0.62) \\ (0.21,0.66) \end{matrix} \end{matrix} \end{matrix} \right]$$

The comprehensive evaluation values being $A_{4}^{1}=(0.6101, 0.2458)$, $A_{4}^{2}=\left( 0.5084, 0.3220 \right)$, $A_{4}^{3}=\left( 0.4072, 0.4302 \right)$

The score function $S\left( A_{4} \right)=(0.2841,0.1832,-0.0273)$.
